# Clinical efficacy of acupuncture for diminished ovarian reserve: a systematic review and meta-analysis of randomized controlled trials

**DOI:** 10.3389/fendo.2023.1136121

**Published:** 2023-08-02

**Authors:** Guangyao Lin, Xiyu Liu, Chao Cong, Siru Chen, Lianwei Xu

**Affiliations:** Department of Gynecology, Longhua Hospital, Shanghai University of Traditional Chinese Medicine, Shanghai, China

**Keywords:** acupuncture, diminished ovarian reserve, randomized controlled trials, meta-analysis, review

## Abstract

**Objective:**

To evaluate the clinical efficacy of acupuncture for the treatment of diminished ovarian reserve (DOR) based on the existing randomized controlled trials (RCTs).

**Methods:**

Nine databases from their inception to December 6th, 2022, were comprehensively searched to retrieve RCTs related to the clinical efficacy of acupuncture for the treatment of DOR. The outcomes of interest were sex hormones level and antral follicle count (AFC). Risk of Bias (RoB) was adopted to assess the quality of the included trials.

**Results:**

A total of 13 RCTs involving 787 patients were included in this meta-analysis. The review of available evidence revealed acupuncture produced a significant efficacy in decreasing follicle-stimulating hormone (FSH) levels (SMD = -1.07, 95%CI [-1.79, -0.36], *p* = 0.003), FSH/LH ratio (MD = -0.31, 95%CI [-0.54, -0.09], *p* = 0.006) and increasing anti-Müllerian hormone (AMH) levels (SMD = 0.25, 95%CI [-0.00, 0.49], *p* = 0.05), along with AFC (MD = 1.87, 95%CI [0.96, 2.79], *p* < 0.0001) compared to controls. Compared with electro-acupuncture treatment, manual acupuncture was superior in reducing FSH levels, FSH/LH ratio, and increasing AMH levels and AFC (*p* < 0.05). A notable association was also seen when acupuncture was combined with traditional Chinese medicine therapy for improving FSH levels, FSH/LH ratio, and AFC (*p* < 0.05). Besides, a high dose of acupuncture (≥10 acupoints) was more conducive to ameliorating FSH levels, FSH/LH ratio, and AFC (*p* < 0.05) than a low dose of acupuncture (<10 acupoints). Substantial heterogeneity existed among studies.

**Conclusion:**

Acupuncture may have significant clinical potential for patients with DOR in terms of improving sex hormones level and increasing AFC, although the evidence is drawn with high heterogeneity. This finding suggests that more rigorous trials conducted in diverse regions worldwide are necessary to identify the efficacy of acupuncture for patients diagnosed with DOR.

**Systematic review registration:**

https://www.crd.york.ac.uk, identifier CRD42023402336.

## 1 Introduction

Diminished ovarian reserve (DOR), manifested as lower fertility due to poor oocyte quality, is a prevalent condition experienced by more than 26% of young patients recently (1, 2). Although the etiology of DOR is currently unclear, an increasing quantity of evidence demonstrated various conditions contributed to the process of DOR, such as higher age (3), natural history (4), surgery (5, 6), chemotherapy (7), as well as lifestyle behaviors, including frequent binge drinking and smoking (8, 9). Furthermore, the accepted definition of DOR is not uniform globally owing to the changing lab testing options and complex interpretation for ovarian reserve tests, but the popular clinical diagnosis for DOR involved follicle-stimulating hormone (FSH), anti-Müllerian hormone (AMH) and antral follicle count (AFC) (10, 11).

At present, the treatment regimen for DOR is based on whether the patient has fertility needs, menstrual dysfunction, and the symptoms of estrogen deficiency. For example, ovulation induction assisted reproductive technology (ART) and coenzyme Q10 were adopted for those with infertility (12–14). And pharmacotherapies such as hormonal contraceptives, estrogen, and progesterone were usually used to restore the menstrual cycle regularity. However, these may account for adverse risks like ovarian hyperstimulation syndrome, venous thromboembolism, stroke, and breast cancer (15, 16). Owing to these side effects, an increasing number of patients with DOR have sought complementary and alternative medicine based on growing clinical evidence, such as acupuncture, to improve treatment outcomes (17, 18).

Acupuncture, a nonpharmacological intervention, has been widely introduced in the reproductive disorders field with supportive scientific evidence (19). Leading organizations in the field, such as the Chinese Reproductive Medicine Group, recommend acupuncture for strengthening ovarian reserve (20). Recently, randomized controlled trials (RCTs) on acupuncture for DOR have been increasing, but these clinical studies have usually had small sample sizes, and therefore the findings have been inconsistent (21). Hence, we conducted a systematic review and meta-analysis of the existing evidence with critical evaluation to inform clinical practice. The investigation’s specific objective was as follows: Is acupuncture effective in regulating sex hormones level and increasing AFC in patients with DOR?

## 2 Materials and methods

This study was conducted following the preferred reporting program of the systematic review and meta-analysis (PRISMA) (22), and was registered on PROSPERO (registration number: CRD42023402336).

### 2.1 Search strategy

Six English-language databases (Web of Science, Sinomed, EBSCO, Scopus, PubMed, and Cochrane Library) and three Chinese-language databases (China National Knowledge Infrastructure, Wanfang, and VIP Information) were thoroughly searched from inception up to December 6th, 2022, for eligible RCTs. The search strategy is made up of three components: clinical condition (diminished ovarian reserve, declined ovarian reserve, decreased ovarian reserve), intervention (acupuncture, electro-acupuncture, manual acupuncture), and study type (randomized clinical trial). Besides, the references of retrieved studies were evaluated carefully to look for more potentially relevant articles.

### 2.2 Inclusion and exclusion criteria

A relevant RCT satisfied the following inclusion criteria would be included: (1) the patients diagnosed with DOR (FSH≥10IU/L or AMH<1.1ng/mL or AFC<5~7) according to the diagnostic criteria issued by China Expert Group of Consensus on Clinical Diagnosis & Management of Diminished Ovarian Reserve (23); (2) articles were RCTs (with or without blinding) investigated the connection of acupuncture with DOR; (3) eligible interventions were acupuncture, including manual acupuncture and electro-acupuncture regardless of needling techniques; (4) studies reported sex hormones level or related clinical parameters with sufficient data at least. If acupuncture was conducted as an adjunct to treatment for DOR and the same concomitant treatment as the trial group was used by the control group, the studies would be included. Control arms could be Western medications, traditional Chinese medicines, sham acupuncture, wait-list, usual care, or no treatment.

The exclusion criteria were as listed: (1) studies were not acupuncture therapy (e.g., massage, moxibustion, electrostimulation without needles); (2) studies compared with different acupuncture treatments (e.g., acupoint catgut embedding); (3) patients incorporated with other endocrine diseases (e.g., polycystic ovary syndrome, thyroid dysfunction, and hyperprolactinemia); (4) patients suffered from DOR due to radiation therapy or chemotherapy; (5) studies were duplicate publications, reviews, meta-analysis, study protocols, and animal experiments; (6) studies were not published in the Chinese or English language.

### 2.3 Data extraction and risk of bias

According to the aforementioned eligibility criteria, all data were extracted independently using predesigned forms. Any disagreements were resolved by consulting the third review author (L.W.X.). Study features (the author’s last name, publication time, and sample capacity), details of the interventions, and the outcomes were extracted from each RCT. In addition, two reviewers (G.Y.L. and C.C.) independently appraised the quality and the risk of bias (RoB) of the studies included. The risk of bias was estimated with RoB 2.0 (24). Each RCT was assigned to 5 specific domains: randomization process, deviations from the established intervention, missing outcome data, measurement of the outcome, and selective outcome reporting. Each domain was rated as low, high, or some concerns. Moreover, we intended to contact the article authors for additional information when needed.

### 2.4 Statistical analysis

EndNote 20.2 software was adopted for data management. Stata 15.1 and Review Manager 5.3 software were performed for statistical analysis. The continuous data were summarized with a standardized mean difference (SMD) or mean difference (MD) with 95% confidence intervals (CIs). The inter-study heterogeneity was assessed with I^2^ statistics. An I^2^ ≤ 50% indicated no statistically significant heterogeneity. The fixed-effect model ought to be applied. Otherwise, the more appropriate random-effect model was adopted. Two-sided *p* ≤ 0.05 was deemed as statistically significant. Subgroup analysis were performed to compare the effectiveness with different interventions in treating DOR and explore potential sources of heterogeneity. Publication bias was evaluated by Begg’s and Egger’s tests when ten studies were included at least.

## 3 Results

### 3.1 Included articles

**Figure 1:**
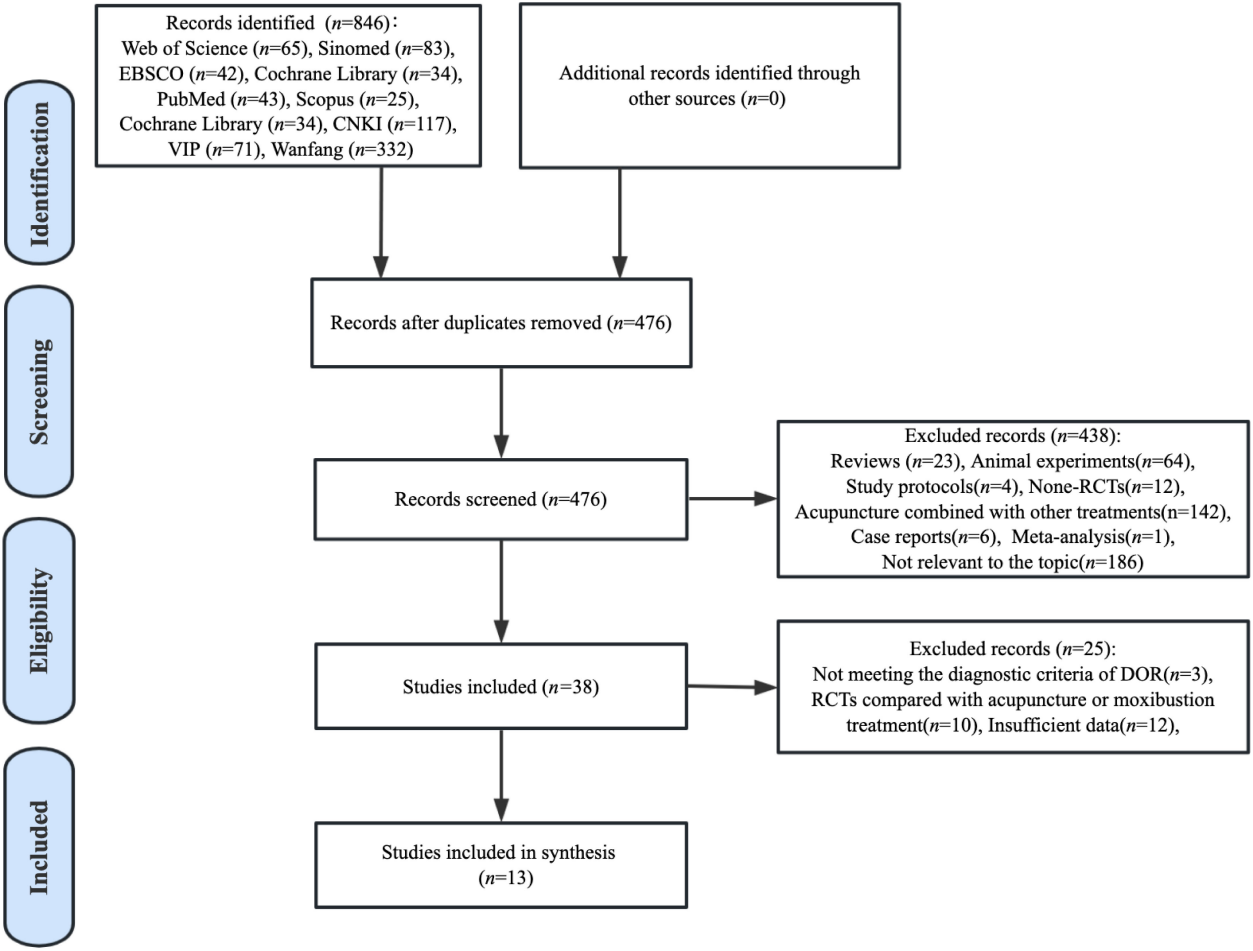
PRISMA flow chart.

A total of 846 articles concerning the clinical efficacy of acupuncture for DOR were identified through preliminary database searches. Among the 846 pieces of literature, 370 duplicate publications were excluded, and 438 papers were removed owing to not fulfilling the inclusion criteria. Then, we carefully removed another 25 studies because they did not have sufficient data to analyze, or did not meet the diagnostic criteria of DOR. Finally, 13 RCTs published between 2015 to 2022 were included in the meta-analysis. The selection flowchart for the included publications is depicted in **Figure 1**.

### 3.2 Study characteristics

**Table 1** summarizes the study characteristics of these RCTs. Quantitative synthesis was conducted with 13 RCTs *via* a meta-analysis by pooling the results. These RCTs’ sample sizes ranged from 40 to 100. A total of 787 patients with DOR were divided into trial group (acupuncture group) and control group with 391 and 396 cases in each group, respectively. All these clinical trials were conducted in China. Among the 13 RCTs included, of which 12 clinical trials treatment duration was three months (25–35, 37), except one trial was two months (36). In addition, electro-acupuncture was adopted in four studies (25, 26, 29, 30) and manual acupuncture was adopted in 9 studies (27, 28, 31–37). Seven studies compared acupuncture with traditional Chinese medicine (28–33, 37); Four studies compared acupuncture with hormone medicine (25, 27, 35, 36); One study compared acupuncture with the combination use of traditional Chinese medicine and hormone medicine (34); One study compared acupuncture with wait-list (26).

**Table 1:** Study characteristics.

Study	Year	Sample size (n)	Age (year)		Disease duration (year)		Antral follicle count		Treatment regimen		Dose of acupuncture	Treatment duration	Outcomes
		T/C	T	C	T	C	T	C	T	C			
Zhao (25)	2021	30/30	33 ± 5	33 ± 5	5.57 ± 1.95	5.65 ± 2.30	4.12 ± 0.62	4.08 ± 0.56	EA	Progynova + Duphaston	9 acupoints	3 months	①②④⑤⑥
Li (A) (26)	2018	20/20	34.30 ± 3.94	35.45 ± 2.84	2.40 ± 0.83	2.14 ± 0.58	4.70 ± 2.89	3.70 ± 3.39	EA	Wait-list	13 acupoints	3 months	①③⑥
Song (27)	2019	40/40	33 ± 4	34 ± 4	1.4 ± 0.3	1.3 ± 0.3	5.47 ± 0.40	5.46 ± 0.42	MA + Climen	Climen	12 acupoints	3 months	①②④⑤⑥
Chai (28)	2018	50/50	35.3 ± 2.6	35.8 ± 2.4	2.9 ± 1.2	3.0 ± 1.0	2.04 ± 1.08	2.08 ± 1.12	MA + TCM	TCM	11 acupoints	3 months	①④⑤⑥
Feng (A) (29)	2020	28/29	32 ± 5	32 ± 6	0.83 ± 0.49	0.9 ± 0.41	3.05 ± 1.05	3.30 ± 1.03	EA + TCM	TCM	8 acupoints	3 months	①②④⑤
Feng (B) (30)	2020	20/20	NA	NA	NA	NA	NA	NA	EA + TCM	Placebo Acup + TCM	7 acupoints	3 months	①②④⑤⑥
Wan (31)	2021	30/30	35 ± 3	33 ± 5	NA	NA	NA	NA	MA + TCM	TCM	11 acupoints	3 months	①②④⑤
Jiang (32)	2022	30/30	35.54 ± 3.32	35.55 ± 3.27	NA	NA	NA	NA	MA	TCM	9 acupoints	3 months	①②③④
Hu (33)	2020	44/44	36.88 ± 3.58	36.91 ± 3.82	3.41 ± 0.78	3.56 ± 0.89	2.06 ± 1.10	2.15 ± 1.19	MA + TCM	TCM	10 acupoints	3 months	①④⑤⑥
Zhang (34)	2020	23/23	35.55 ± 3.30	36.05 ± 1.96	1.83 ± 0.46	1.94 ± 0.52	5.40 ± 1.82	4.90 ± 1.80	MA + TCM +Femoston	TCM + Femoston	7 acupoints	3 months	①③④⑤
Tian (35)	2015	23/23	33.19 ± 4.12	33.86 ± 2.92	NA	NA	NA	NA	MA + Progynova + Progesterone	Progynova + Progesterone	5 acupoints	3 months	①②④
Gou (36)	2019	24/27	34.46 ± 5.32	34.22 ± 4.64	NA	NA	4.25 ± 1.36	4.24 ± 1.69	MA + CC + HMG	CC + HMG	9 acupoints	2 months	③⑥
Li (B) (37)	2018	29/30	30.93 ± 3.75	31.20 ± 3.78	2.04 ± 1.04	1.77 ± 0.99	3.10 ± 1.93	3.00 ± 1.49	MA + TCM	TCM	9 acupoints	3 months	①②③④⑤⑥

T, trial group; C, control group; EA, electro-acupuncture; MA, manual acupuncture; NA, not available; CC, clomiphene citrate; HMG, human menopausal gonadotrophin; TCM, traditional Chinese medicine; ① follicle stimulating hormone (FSH); ② luteinizing hormone (LH); ③ anti-Müllerian hormone (AMH); ④ estradiol (E_2_); ⑤ FSH/LH ratio; ⑥ antral follicle count (AFC).

### 3.3 Risk of bias

**Figure 2:**
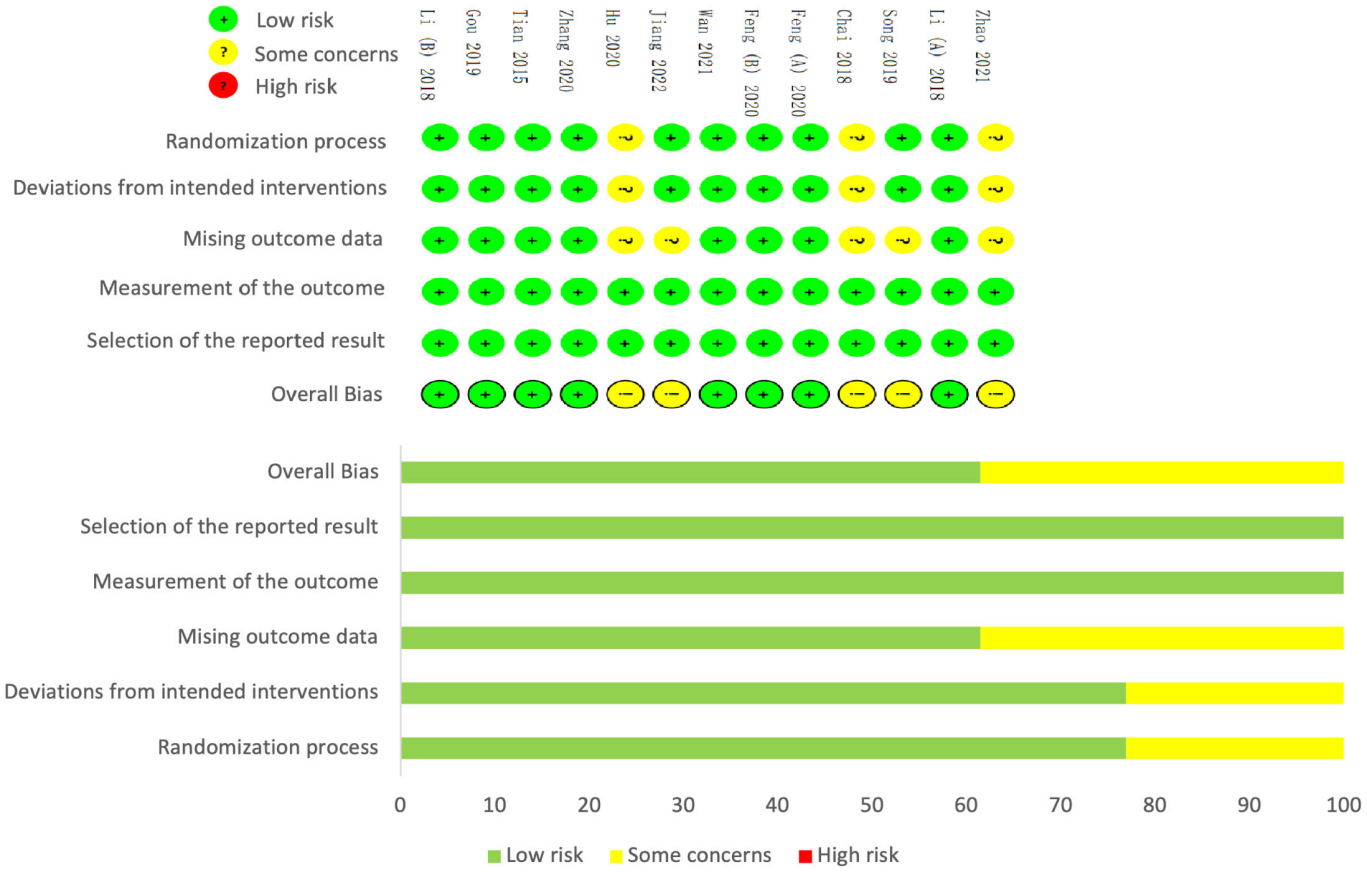
Risk of bias assessment.

Overall, five studies were assessed to have some concerns about the risk of bias. The methodological quality of nine documents (26, 27, 29–32, 34–36) provided a detailed procedure of how patients were randomized. Three (25, 28, 33) out of 13 studies were judged to have some concerns about the risk of bias in the randomization process domain principally because they failed to report how a random sequence was generated or just described of “random” assignment. In addition, they did not take appropriate analysis or provide adequate information on the analysis strategies. Moreover, five studies (25, 27, 28, 32, 33) were judged to have some concerns about the risk of bias in missing outcome data, as incomplete outcomes information was not reported, which implied the relation to the true values of the missing outcome data. (**Figure 2**).

### 3.4 Outcome measurements

#### 3.4.1 Sex hormones level

**Figure 3:**
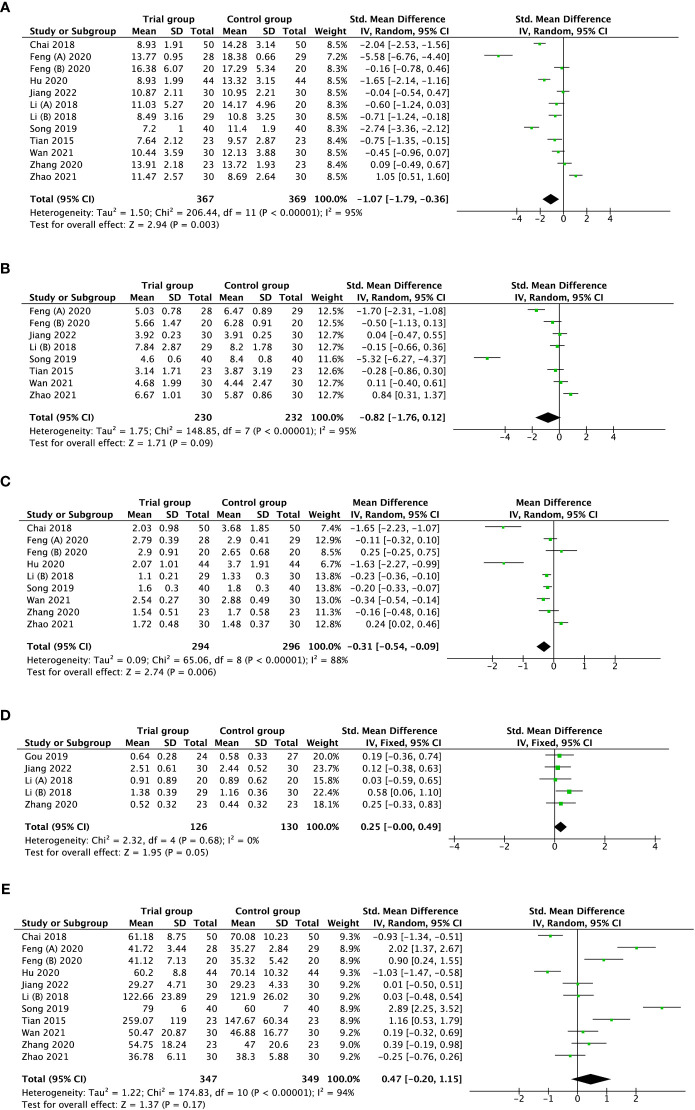
Forest plot of the correlation of acupuncture therapy with hormone levels, FSH levels **(A)**; LH levels **(B)**; FSH/LH ratio **(C)**; AMH levels **(D)**; E_2_ levels **(E)**.

**Figure 4:**
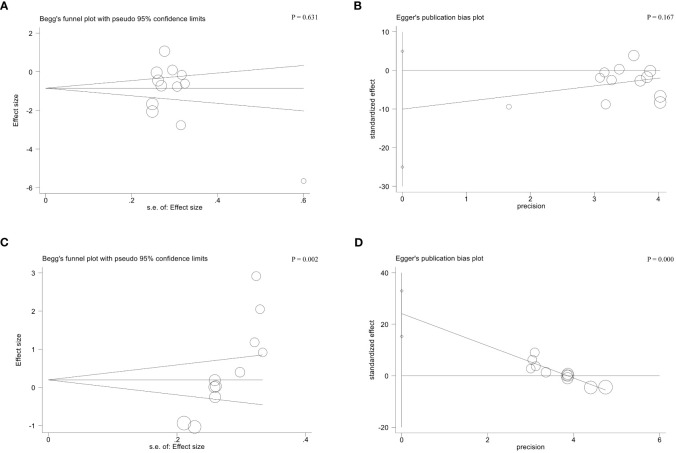
Begg’s test **(A)** and Egger’s test **(B)** for FSH levels; Begg’s test **(C)** and Egger’s test **(D)** for E_2_ levels.

Regarding hormones level (**Figure 3**), pooled results demonstrated a significant decrease in FSH levels (SMD = -1.07, 95%CI [-1.79, -0.36], I^2^ =95, *p* = 0.003, **Figure 3A**), FSH/LH ratio (MD = -0.31, 95%CI [-0.54, -0.09], I^2^ =88, *p* = 0.006, **Figure 3C**), and increase in AMH levels (SMD = 0.25, 95%CI [-0.00, 0.49], I^2^ =0, *p* = 0.05, **Figure 3D**) in the trial group (acupuncture treatment) compared with the control group who received traditional Chinese medicine and/or hormone medicine or no treatment. Nevertheless, LH levels (SMD = -0.82, 95%CI [-1.76, 0.12], I^2^ =95, *p* = 0.09, **Figure 3B**), and E_2_ levels (SMD = 0.47, 95%CI [-0.20, 1.15], I^2^ =94, *p* = 0.17, **Figure 3E**) were not improved notably after acupuncture therapy. Because there was substantial heterogeneity existing in the outcome of FSH, LH, FSH/LH ratio, and E_2_, the random-effect model was applied. And the fixed-effect model was adopted for the outcome of AMH owing to no heterogeneity. As for the publication bias among included studies, no apparent asymmetry was observed based on Begg’s and Egger’s tests for FSH levels (*p* > 0.05); still, there was significant publication bias for E_2_ levels (*p* < 0.05) (**Figure 4**).

#### 3.4.2 Antral follicle count

**Figure 5:**
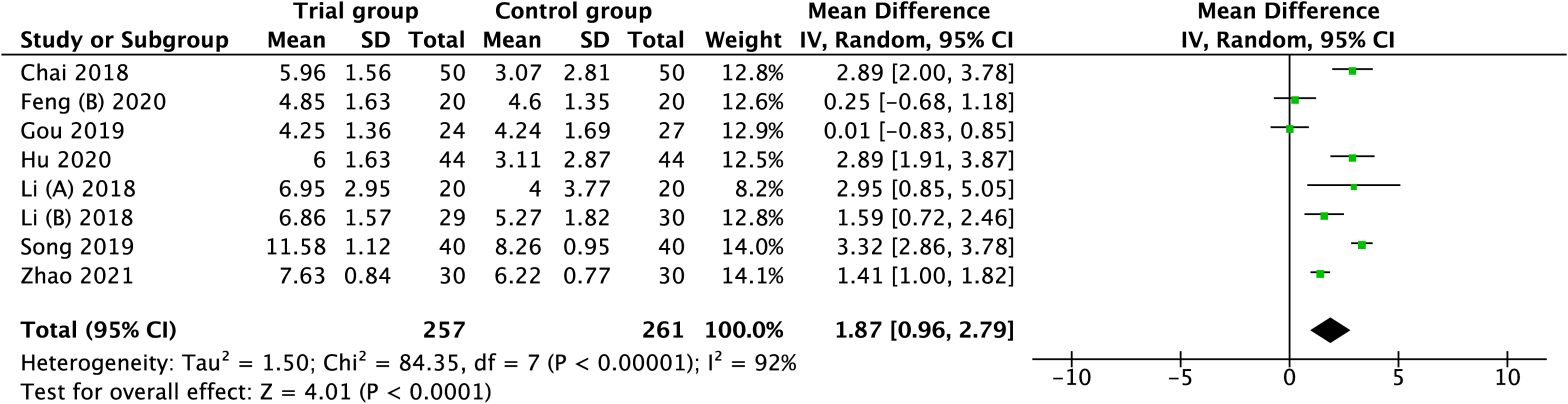
Forest plot of the correlation of acupuncture therapy with antral follicle count.

A total of eight studies involving 518 patients were extracted into this meta-analysis for the outcome of AFC. As there was considerable heterogeneity existing among these studies(I^2^=92), the random-effect model was applied, and the pooled result revealed that the increase in AFC was associated with acupuncture when compared with none-acupuncture therapy (MD = 1.87, 95%CI [0.96, 2.79], I^2^ =92, *p* < 0.0001), (**Figure 5**).

#### 3.4.3 Subgroup analysis

In the subgroup analysis for different types of intervention, the pooled result favored electro-acupuncture therapy in increasing AFC (*p* < 0.05). Studies that used manual acupuncture showed significant efficacy in improving FSH levels, FSH/LH ratio, AMH levels, and AFC (*p* < 0.05). As for the intervention of acupuncture or acupuncture plus traditional Chinese medicine also significantly decreased FSH levels and FSH/LH ratio, along with increased AFC (*p* < 0.05). Acupuncture or acupuncture plus Western medicine was not favorable to improving hormones level and AFC (*p* > 0.05). In addition, a high dose of acupuncture (≥10 acupoints) was conducive to ameliorating FSH levels, FSH/LH ratio, and AFC (*p* < 0.05). A low dose of acupuncture (<10 acupoints) was also a significant modifier of AMH and E_2_ levels. **Table 2** shows the results of the subgroup analysis of the correlation of different types of intervention with hormones level and AFC.

**Table 2:** The subgroup analysis of the correlation of acupuncture with hormones level and antral follicle count.

Type of Intervention	Study (n)	Case (n)	SMD/MD 95% CI	*p*	I^2^ (%)	Model
Electro-acupuncture						
FSH	4	197	-1.25 [-3.24, 0.74]	0.22	97	Random
LH	3	157	-0.44 [-1.93, 1.04]	0.56	95	Random
FSH/LH ratio	3	157	0.10 [-0.17, 0.37]	0.47	65	Random
AMH	1	40	0.03 [-0.59, 0.65]	0.94	NA	Random
E_2_	3	157	0.88 [-0.46, 2.22]	0.20	93	Random
AFC	3	140	1.26 [0.17, 2.34]	0.02	74	Random
Manual acupuncture						
FSH	8	539	-1.03 [-1.71, -0.36]	0.003	92	Random
LH	5	305	-1.07 [-2.42, 0.29]	0.12	96	Random
FSH/LH ratio	6	433	-0.53 [-0.80, -0.26]	0.0001	88	Random
AMH	4	216	0.29 [0.02, 0.56]	0.04	0	Random
E_2_	8	539	0.32 [-0.48, 1.12]	0.43	95	Random
AFC	5	378	2.15 [0.91, 3.39]	0.0007	92	Random
Acupuncture plus TCM						
FSH	6	404	-1.60 [-2.66, -0.55]	0.003	95	Random
LH	5	276	-0.59 [-1.23, 0.06]	0.08	85	Random
FSH/LH ratio	6	404	-0.52 [-0.99, -0.06]	0.03	80	Random
AMH	2	119	0.35 [-0.10, 0.79]	0.13	34	Random
E_2_	7	464	0.35 [-0.50, 1.20]	0.41	95	Random
AFC	4	287	0.93 [0.49, 1.38]	<0.00001	68	Random
Acupuncture plus WM						
FSH	3	186	-0.81 [-2.97, 1.36]	0.46	98	Random
LH	3	186	-1.56 [-4.54, 1.42]	0.31	98	Random
FSH/LH ratio	2	140	-0.06 [-1.25, 1.13]	0.92	92	Random
AMH	1	51	0.19 [-0.36, 0.74]	0.49	NA	Random
E_2_	3	186	1.26 [-0.56, 3.08]	0.18	97	Random
AFC	3	191	1.63 [-0.16, 3.41]	0.07	96	Random
High dose of acupuncture (≥10 acupoints)						
FSH	5	368	-1.50 [-2.30, -0.69]	0.0003	91	Random
LH	2	140	-2.59 [-7.91, 2.73]	0.34	99	Random
FSH/LH ratio	4	328	-0.93 [-1.16, -0.70]	<0.00001	0	Random
AMH	1	40	0.03 [-0.59, 0.65]	0.94	NA	Random
E_2_	4	328	0.26 [-1.27, 1.80]	0.74	97	Random
AFC	4	308	1.89 [1.05, 2.72]	<0.00001	89	Random
Low dose of acupuncture (<10 acupoints)						
FSH	7	358	-0.77 [-1.76, 0.21]	0.12	94	Random
LH	6	322	-0.28 [-0.92, 0.36]	0.39	87	Random
FSH/LH ratio	5	262	-0.12 [-0.62, 0.39]	0.65	76	Random
AMH	4	216	0.29 [0.02, 0.56]	0.04	0	Random
E_2_	7	368	0.59 [0.02, 1.16]	0.04	86	Random
AFC	4	210	0.70 [-0.06, 1.46]	0.07	86	Random

HR, hazard ratio; CI, confidence interval; NA, not available; TCM, traditional Chinese medicine; WM, Western medicine.

## 4 Discussion

Acupuncture, as a novel therapy for DOR (38), has been proven to involve diverse cellular functions and mechanisms. Zhang et al. (39) found that electro-acupuncture could increase primordial follicle counts, E_2_ and AMH levels, while decreasing FSH and LH levels by targeting the PI3K/AKT/mTOR signaling pathway. Another study indicated that acupuncture could inhibit bta-miR-7857-3p_R-1, mdo-miR-26b-5p_R+1_1ss10TC, and rno-miR-92b-3p expression in ovarian tissues and improve ovarian function *via* relieving DOR-mediated oxidative stress (18). Besides, some investigations also found acupuncture could reduce granulosa cell autophagy by weakening the expression of LncMEG3 and regulating the PI3K/AKT/mTOR pathway (40). The findings were consistent with Wang et al. (41). Interestingly enough, electro-acupuncture could alter the intestinal microbiota and block the accumulation of Fe^2+^, thereby increasing mature follicles as well as improving the sex hormones level in premature ovarian failure mice (42). Moreover, a clinical trial published recently demonstrated that electro-acupuncture might improve embryonic development and oocyte quality by regulating IRS-1/PI3K/GLUT4 pathway in ovarian granulosa cells (43). Similarly, Kim et al. (44) revealed that acupuncture combined with *in vitro* fertilization (IVF) therapy remarkably enhanced the number of retrieved mature oocytes, regardless of controlled ovarian hyperstimulation cycles, compared with the IVF therapy alone in women aged > 37 years.

To the best of our knowledge, this is the first meta-analysis to evaluate the clinical efficacy of acupuncture for DOR. In this study, we included 13 RCTs involving 787 patients to investigate the association of acupuncture therapy with DOR. Evidence was found that the use of acupuncture was correlated with decreased FSH levels, FSH/LH ratio, and increased AFC and AMH levels. According to our results, the LH and E_2_ levels could not be statistically significantly improved with acupuncture intervention. Moreover, to provide more convincing evidence and explore the potential factors that may affect the clinical efficacy of acupuncture, a subgroup analysis based on the different types of intervention was conducted. The results indicated manual acupuncture was superior in reducing FSH levels, FSH/LH ratio, and increasing AMH levels and AFC when compared with electro-acupuncture treatment. Furthermore, a notable association was also seen when acupuncture was combined with traditional Chinese medicine therapy for improving FSH levels, FSH/LH ratio, and AFC. Besides, a high dose of acupuncture (≥10 acupoints) was more conducive to ameliorating FSH levels, FSH/LH ratio, and AFC than a low dose of acupuncture (<10 acupoints). Nevertheless, our subgroup analysis included a limited number of RCTs. For example, only one study was included when estimating the correlation of AMH levels with different types of intervention (electro-acupuncture, acupuncture plus Western medicine, and high dose of acupuncture), and it produced no convincing results. This revealed that our result might be more probably on account of insufficient statistical power rather than due to a lack of clinical efficacy from acupuncture.

While this meta-analysis results, from a clinical perspective, support the treatment with acupuncture in patients with DOR, it should be considered that these findings were derived from 13 RCTs with high heterogeneity. To minimize heterogeneity, we performed subgroup analysis based on six different types of intervention, but the heterogeneity was stable. The underlying factors contributed to severe heterogeneity were as followings: first, all included studies were single-center trials; thus, the adjunctive regimen with acupuncture varied considerably among studies. For instance, although the adjunctive regimens were traditional Chinese medicine, herb types and doses within each study were various. Second, acupuncture acupoints and examination technology adopted by included studies were not uniform, which likely contributed to heterogeneity as well. Furthermore, pregnancy outcomes are one of the major concerns for reproductive-aged women with DOR, but only one study (36) reported this. Therefore, we failed to explore the correlation of acupuncture with pregnancy outcomes, which might be an inherent deficiency of our meta-analysis. Besides, the 13 RCTs were from China, which usually incorporates poor descriptions of their methodologies, such as only two studies included reported blinding. Nevertheless, nonblinded pragmatic trials which emphasize extrapolation and practical applicability in real-world situations, in recent years, have been recommended to acquire clinically connected outcomes over treatment efficacy (45). This suggestion is especially qualified for estimating flexible and complicated interventions, such as acupuncture (46). To guarantee the quality of source RCTs, we adopted more stringent inclusion criteria, and a substantial endeavor was made to perform an extensive literature search. Consequently, we can only present a weak proposal to access acupuncture therapy as part of comprehensive DOR management. However, further investigations including larger and higher-quality RCTs are warranted to reinforce or refute the current evidence.

## 5 Conclusion

These findings suggest that acupuncture, as a nonpharmacological intervention, has excellent clinical potential for patients with DOR in decreasing FSH levels and FSH/LH ratio, along with increasing AMH levels and AFC. Acupuncture may be recommended for the treatment of DOR. However, our findings should be cautiously adopted due to the high heterogeneity. Therefore, more high-quality studies conducted in diverse regions worldwide are necessary to verify the efficacy of acupuncture for patients diagnosed with DOR.

## Data availability statement

The original contributions presented in the study are included in the article. Further inquiries can be directed to the corresponding author.

## Author contributions

Study design: GYL, LWX. Data collections: GYL, SRC, CC. Data analysis: GYL, CC, XYL. Writing the manuscript: GYL. Revising the manuscript: LWX. All authors read and approved the final version of manuscript.

## References

[1] Steiner AZ Pritchard D Stanczyk FZ Kesner JS Meadows JW Herring AH . Association between biomarkers of ovarian reserve and infertility among older women of reproductive age. JAMA (2017) 318(14):1367–76. doi: 10.1001/jama.2017.14588 PMC5744252 29049585

[2] Devine K Mumford SL Wu M DeCherney AH Hill MJ Propst A . Diminished ovarian reserve in the United States assisted reproductive technology population: diagnostic trends among 181,536 cycles from the Society for Assisted Reproductive Technology Clinic Outcomes Reporting System. Fertil Steril (2015) 104(3):612–19.e3. doi: 10.1016/j.fertnstert.2015.05.017 PMC4560955 26049057

[3] Jaswa EG McCulloch CE Simbulan R Cedars MI Rosen MP . Diminished ovarian reserve is associated with reduced euploid rates *via* preimplantation genetic testing for aneuploidy independently from age: evidence for concomitant reduction in oocyte quality with quantity. Fertil Steril (2021) 115(4):966–73. doi: 10.1016/j.fertnstert.2020.10.051 33583594

[4] Lew R . Natural history of ovarian function including assessment of ovarian reserve and premature ovarian failure. Best Pract Res Clin Obstet Gynaeco (2019) 55:2–13. doi: 10.1016/j.bpobgyn.2018.05.005 30420162

[5] Goodman LR Goldberg JM Flyckt RL Gupta M Harwalker J Falcone T . Effect of surgery on ovarian reserve in women with endometriomas, endometriosis and controls. Am J Obstet Gynecol (2016) 215(5):589.e1–6. doi: 10.1016/j.ajog.2016.05.029 27242204

[6] Ganer Herman H Gluck O Keidar R Kerner R Kovo M Levran D . Ovarian reserve following cesarean section with salpingectomy vs tubal ligation: a randomized trial. Am J Obstet Gynecol (2017) 217(4):472.e1–6. doi: 10.1016/j.ajog.2017.04.028 28455082

[7] Spears N Lopes F Stefansdottir A Rossi V De Felici M Anderson RA . Ovarian damage from chemotherapy and current approaches to its protection. Hum Reprod Update (2019) 25(6):673–93. doi: 10.1093/humupd/dmz027 PMC6847836 31600388

[8] Hawkins Bressler L Bernardi LA De Chavez PJD Baird DD Carnethon MR Marsh EE . Alcohol, cigarette smoking, and ovarian reserve in reproductive-age African-American women. Am J Obstet Gynecol (2016) 215(6):758.e1–9. doi: 10.1016/j.ajog.2016.07.012 PMC5124512 27418446

[9] de Angelis C Nardone A Garifalos F Pivonello C Sansone A Conforti A . Smoke, alcohol and drug addiction and female fertility. Reprod Biol Endocrinol (2020) 18(1):21. doi: 10.1186/s12958-020-0567-7 32164734 PMC7069005

[10] Pastore LM Christianson MS Stelling J Kearns WG Segars JH . Reproductive ovarian testing and the alphabet soup of diagnoses: DOR, POI, POF, POR, and FOR. J Assist Reprod Genet (2018) 35(1):17–23. doi: 10.1007/s10815-017-1058-4 28971280 PMC5758472

[11] Practice Committee of the American Society for Reproductive Medicine Practice Committee of the American Society for Reproductive Medicine . Testing and interpreting measures of ovarian reserve: a committee opinion. Fertil Steril (2020) 114(6):1151–7. doi: 10.1016/j.fertnstert.2020.09.134 33280722

[12] Xu Y Nisenblat V Lu C Li R Qiao J Zhen X . Pretreatment with coenzyme Q10 improves ovarian response and embryo quality in low-prognosis young women with decreased ovarian reserve: a randomized controlled trial. Reprod Biol Endocrinol (2018) 16(1):29. doi: 10.1186/s12958-018-0343-0 29587861 PMC5870379

[13] Chen L Lai M Huang X Zeng F Xia W Yang Q . Clinical efficacy of assisted reproductive technology combined with progesterone capsules in the treatment of infertility caused by diminished ovarian reserve and its influence on serum FSH, E, and LH levels of patients. J Healthc Eng (2022) 2022:5319172. doi: 10.1155/2022/5319172 35368963 PMC8970857

[14] Tu X You B Jing M Lin C Zhang R . Progestin-primed ovarian stimulation versus mild stimulation protocol in advanced age women with diminished ovarian reserve undergoing their first fertilization cycle: A retrospective cohort study. Front Endocrinol (Lausanne) (2021) 12:801026. doi: 10.3389/fendo.2021.801026 35140685 PMC8818948

[15] Wei D Liu J-Y Sun Y Shi Y Zhang B Liu JQ . Frozen versus fresh single blastocyst transfer in ovulatory women: a multicentre, randomised controlled trial. Lancet (2019) 393(10178):1310–8. doi: 10.1016/S0140-6736(18)32843-5 30827784

[16] Deligdisch-Schor L . Hormone therapy effects on the uterus. Adv Exp Med Biol (2020) 1242:145–77. doi: 10.1007/978-3-030-38474-6_8 32406032

[17] Jang S Kim KH Jun JH You S . Acupuncture for *in vitro* fertilization in women with poor ovarian response: a systematic review. Integr Med Res (2020) 9(2):100395. doi: 10.1016/j.imr.2020.02.003 32322482 PMC7160570

[18] Lu G Zhu Y-Y Li H-X Yin Y-L Shen J Shen M-H . Effects of acupuncture treatment on microRNAs expression in ovarian tissues from Tripterygium glycoside-induced diminished ovarian reserve rats. Front Genet (2022) 13:968711. doi: 10.3389/fgene.2022.968711 36212128 PMC9532950

[19] Fu H Sun J Tan Y Zhou H Xu W Zhou J . Effects of acupuncture on the levels of serum estradiol and pituitary estrogen receptor beta in a rat model of induced super ovulation. Life Sci (2018) 197:109–13. doi: 10.1016/j.lfs.2018.02.005 29421437

[20] Qu F Li R Sun W Lin G Zhang R Yang J . Use of electroacupuncture and transcutaneous electrical acupoint stimulation in reproductive medicine: a group consensus. J Zhejiang Univ Sci (2017) 18(3):186–93. doi: 10.1631/jzus.B1600437 PMC5369245 28271655

[21] Ko JH Kim S-N . A literature review of women's sex hormone changes by acupuncture treatment: analysis of human and animal studies. Evidence-Based Complementary Altern Med ECAM (2018) 2018:3752723. doi: 10.1155/2018/3752723 PMC6276442 30581481

[22] Moher D Liberati A Tetzlaff J Altman DG . Preferred reporting items for systematic reviews and meta-analyses: the PRISMA statement. BMJ (2009) 339:b2535. doi: 10.1136/bmj.b2535 19622551 PMC2714657

[23] Expert Group of Consensus on Clinical Diagnosis & Management of Diminished Ovarian Reserve Reproductive Endocrinology & Fertility Preservation Section of Chinese Society on Fertility Preservation under Chinese Preventive Medicine Association . Consensus on clinical diagnosis and management of diminished ovarian reserve. J Reprod Med (2022) 31(4):425–34. doi: 10.3969/j.issn.1004-3845.2022.04.001

[24] Sterne JAC Savović J Page MJ Elbers RG Blencowe NS Boutron I . RoB 2: a revised tool for assessing risk of bias in randomised trials. BMJ (2019) 366:l4898. doi: 10.1136/bmj.l4898 31462531

[25] Zhao S Jing Q Zhengyan Z Dandan S Guigang Z . Effects of electroacupuncture on sex hormone levels and ovarian reserve function in patients with decreased ovarian function. Shanghai J Acupuncture Moxibustion (2021) 40(6):721–6. doi: 10.13460/j.issn.1005-0957.2021.13.0028

[26] Li X Xu H Liu B Yang H Shang J Fang Y . 'Tiaojing Cuyun' acupuncture therapy in diminished ovarian reserve: A randomized controlled trial. China J Tradit Chin Med Pharm (2018) 33(5):1736–9.

[27] Song M Sheng X . Effect of acupuncture on ovarian function and utero-ovarian blood flow index in patients with renal deficiency and liver depression with reduced ovarian reserve function. Chin Remedies Clinics (2019) 19(17):2987–90. doi: 10.11655/zgywylc2019.17.048

[28] Chai H . Clinical observation on the treatment of decreased ovarian reserve function in kidney deficiency type by combining the method of tonifying the kidney and regulating the circumference with acupuncture. Chin J Ethnomed Ethnopharm (2018) 27(11):88–9. doi: 10.3969/j.issn.1007-8517.2018.11.zgmzmjyyzz201811030

[29] Feng X Jia Z Li N Jiang S Chang Z Zhu H . Effect of electroacupuncture combined with Yuyin pill on sex hormone and Th2 cytokines in patients of decreased ovarian reserve function with liver-kidney yin deficiency. Chin Acupuncture Moxibustion (2020) 40(9):959–63. doi: 10.13703/j.0255-2930.20190925-0003 32959591

[30] Feng X Gu Y Zhao Y Wang W . Clinical observation on acupuncture combined with yuyin pills in treating patients with decreased ovarian reserve and changes of ovarian ultrasonogram. J Guangzhou Univ Tradit Chin Med (2020) 37(9):1684–9. doi: 10.13359/j.cnki.gzxbtcm.2020.09.012

[31] Wan Y Yu S . Efficacy observation of acupuncture combined with chinese medication for decreased ovarian reserve. Shanghai J Acupuncture Moxibustion (2021) 40(5):546–50. doi: 10.13460/j.issn.1005-0957.2021.05.0546

[32] Jiang Z Ye C Wang H . Observation on the clinical efficacy of acupuncture and medicine combined with sequential therapy for the treatment of ovarian reserve dysfunction. Proc Clin Med (2022) 31(7):498–500. doi: 10.16047/j.cnki.cn14-1300/r.2022.07.002

[33] Hu Y . Clinical study on the treatment of ovarian reserve function decline in the kidney deficiency type by combining Chinese herbal medicine to tonify the kidney and regulate the circumference method with acupuncture. J Pract Tradit Chin Med (2020) 36(6):693–4.

[34] Zhang N . Clinical study on the decline of ovarian reserve function due to kidney deficiency by combined acupuncture and traditional Chinese medicine. Shanxi, China: Shanxi University of Traditional Chinese Medicine (2020).

[35] Tian M . Effect observation and mechanism discussion on the treatment of diminished ovarian reserve by acupuncture. Shandong, China: Shandong University of Traditional Chinese Medicine (2018).

[36] Gou W . A Randomized Controlled Trial of Acupuncture in the Treatment of IVF-ET in Patients with DOR Infertility. Gansu, China: Lanzhou University (2019).

[37] Li W . Clinical Research of Combined Modified Yulinzhu with Abdominal Acupuncture on Treating Diminished Ovarian Reserve. Guangxi, China: Guangxi University of Traditional Chinese Medicine (2018).

[38] Yang L Zhang J Zhang Z Xu S . Improvement effect of acpuncture at Chong and Conception Channels on sex hormones in patients with diminished ovarian reserve. China Med Herald (2019) 16(24):157–61.

[39] Zhang H Qin F Liu A Sun Q Wang Q Xie S . Electro-acupuncture attenuates the mice premature ovarian failure *via* mediating PI3K/AKT/mTOR pathway. Life Sci (2019) 217:169–75. doi: 10.1016/j.lfs.2018.11.059 30521869

[40] Chen X Tang H Liang Y Wu P Xie L Ding Y . Acupuncture regulates the autophagy of ovarian granulosa cells in polycystic ovarian syndrome ovulation disorder by inhibiting the PI3K/AKT/mTOR pathway through LncMEG3. BioMed Pharmacother (2021) 144:112288. doi: 10.1016/j.biopha.2021.112288 34653763

[41] Wang S Lin S Zhu M Li C Chen S Pu L . Acupuncture reduces apoptosis of granulosa cells in rats with premature ovarian failure *via* restoring the PI3K/akt signaling pathway. Int J Mol Sci (2019) 20(24):6311. doi: 10.3390/ijms20246311 31847241 PMC6940951

[42] Geng Z Nie X Ling L Li B Liu P Yuan L . Electroacupuncture may inhibit oxidative stress of premature ovarian failure mice by regulating intestinal microbiota. Oxid Med Cell Longev (2022) 2022:4362317. doi: 10.1155/2022/4362317 36082082 PMC9448555

[43] Xiang S Xia M-F Song J-Y Liu D-Q Lian F . Effect of electro-acupuncture on expression of IRS-1/PI3K/GLUT4 pathway in ovarian granulosa cells of infertile patients with polycystic ovary syndrome-insulin resistance of phlegm-dampness syndrome. Chin J Integr Med (2021) 27(5):330–5. doi: 10.1007/s11655-020-3219-z 32572779

[44] Kim J Lee H Choi TY Kim JI Kang BK Lee MS . Acupuncture for poor ovarian response: A randomized controlled trial. J Clin Med (2021) 10(10). doi: 10.3390/jcm10102182 PMC8158119 34070086

[45] Sox HC Lewis RJ . Pragmatic trials: practical answers to "Real world" Questions. JAMA (2016) 316(11):1205–6. doi: 10.1001/jama.2016.11409 27654606

[46] Ford I Norrie J . Pragmatic trials. N Engl J Med (2016) 375(5):454–63. doi: 10.1056/NEJMra1510059 27518663

